# Development of an ICF-based eligibility procedure for education in Switzerland

**DOI:** 10.1186/1471-2458-11-S4-S7

**Published:** 2011-05-31

**Authors:** Judith Hollenweger

**Affiliations:** 1 Pädagogische Hochschule Zürich, Departement Forschung und Entwicklung Waltersbachstrasse 5, CH-8090, Zürich, Switzerland

## Abstract

Starting in January 2011, Switzerland will implement a multidimensional, context-sensitive procedure to establish eligibility in education systems. This paper provides a brief overview of the different eligibility-related practices with a special focus on children with disabilities. The paper then outlines the philosophical and conceptual framework of the eligibility procedure based on the International Classification of Functioning, Disability and Health, and the UN Convention on the Rights of Persons with Disability. The different components and methodology applied to organise information in the process towards establishing eligibility are also presented. Finally, some observations are made regarding transparent and just applications of the eligibility procedure, and the implementation of this new eligibility procedure.

## Background

Starting in January 2011, Switzerland will be among the first countries to implement a multidimensional, context-sensitive procedure to establish eligibility in education systems. This new eligibility procedure is based on the International Classification of Functioning, Disability and Health (ICF) [1], the Children and Youth Version (ICF-CY) [2]. In addition, it seeks to implement the principles of the UN Convention on the Rights of Persons with Disability (CRPD). Already prior to the publication of the ICF in 2001, practitioners and researchers had agreed that an exclusive focus on impairments or diseases is neither desirable nor constructive in the context of planning for participation and creating opportunities in education systems. It was also agreed that to do justice to the CRPD, the focus should be as much on requirements for changes in the environment as on specialised services offered to an individual child.

A network of professionals interested in the application of the ICF-CY had been working for some years to link school-based and state-level practices relevant for children with disability to the philosophy of the ICF. Participation in two European projects, MHADIE (Measuring Health and Disability in Europe) and MURINET (Multidisciplinary Network on Health and Disability in Europe), was instrumental in developing the necessary expertise. This new eligibility procedure is not the first application of the ICF in the education systems in Switzerland. ICF-based tools and procedures have already been introduced in some schools at local and cantonal levels [3]. These implementation processes have provided the expertise necessary to embark on the complexities of establishing eligibility.

Until 2008, financing special education was a shared responsibility of federal, cantonal and municipal authorities and eligibility was established on the basis of a list of conditions and impairments provided by the Federal Disability Insurance. It was generally agreed that diverse identification rates across Cantons and municipalities reflected diversity in practices rather than student population, giving rise to concerns regarding the equity of distribution of funds. And because eligibility was seen mainly as eligibility to special schools, an increase in identification rates coincided with a higher segregation rate – despite contrary efforts to include more children in regular classes. The national statistics showed different segregation rates across Cantons but provided no further information helpful to understand these differences.

The opportunity to change the existing eligibility scheme arose as a result of the reform of financial perequation and the repartition of tasks between the Federation and the 26 Cantons. Prior to 2008, when this responsibility was handed over to the Cantons, the task of financing specialised services for children with disabilities was shared by the Federation and the Cantons. Compulsory education is traditionally the responsibility of the Cantons; this reform therefore enabled the Cantons to develop a more consistent and integrative approach to establish eligibility. The Cantons mandated their coordinating body, the Swiss Conference of the Cantonal Ministers of Education, to draft an Intercantonal Agreement for Special Needs Education which was subsequently agreed-upon. The agreement stipulates the development of standards and tools to ensure a coherent and equitable system across the 26 Cantons. It will be implemented when a minimum of 10 Cantons vote for its introduction and thereby will become legally binding in those Cantons. The new eligibility procedure is one of these tools and was developed over the last few years. Since the 10th Canton endorsed the Intercantonal Agreement recently, the procedure will be implemented in all the signatory Cantons starting by January 2011.

Within this context, the author and a team of colleagues from the German and French regions of Switzerland were mandated to develop a new eligibility procedure. Early on in the process, a broad discussion on guiding principles was initiated with all stakeholders to ensure compatibility with existing policies and practices. This paper will provide a short overview of the different eligibility-related practices with a special focus on children with disabilities. The philosophical and conceptual framework of the eligibility procedure based on the ICF and the CRPD will be outlined. In addition, the different components and the methodology applied to organise information in the process towards establishing eligibility will be presented. Finally, some procedural considerations with regard to transparent and just applications of the eligibility procedure will be described and some consideration will be given to the implementation of this new eligibility procedure.

## Methods

### Understanding eligibility

Public or private bodies establish “eligibility” to provide access to resources otherwise unavailable to or not needed by the general public. By applying specific criteria and procedures, a person is identified as belonging to a pre-defined group “eligible to benefit”. Certain resources or programmes are believed to be essential to ensure positive outcomes, such as better health, limitation of impact of diseases or impairments on daily life or improvement of participation in specific areas of life. In the context of health problems and disability, meeting specific eligibility criteria may result in access to drugs or treatment [4], access to health insurance [5,6], access to assistive technology [7], admission to a programme [8,9] or access to specialised interventions in education systems [10]. For this purpose, individuals are identified as having special health care needs [11], special support needs in a given setting [12] or belonging to a special group requiring specialised interventions and environments [13].

Eligibility criteria serve also as gate-keepers by granting access to some but denying services and goods to others. A person may be considered to benefit from but not sufficiently in need of a service. Someone else may be considered eligible based on disability status and potential benefit, but not poor enough to receive it at no costs. Therefore, to gain eligibility to some specialised services, eligibility is based upon disability or need and economic status [14,15]. Effective and equitable gate-keeping mechanisms ensure that scarce resources are rationed to meet the needs of those who most require services. More recently, they are also considered an important instrument to help reduce the exclusion of children from education and family settings, e.g. by reducing referral of vulnerable children into institutional care in Eastern European countries [16]. In addition, some services provided by the state are not necessarily valued or socially acceptable; therefore, being identified as eligible is not viewed as something positive. This is the case for services provided mainly for persons with low socio-economic status or minority background. Such groups are over-represented in some special education settings, suggesting that factors other than impairment play a role in determining eligibility [17-19].

Eligibility criteria are complex social constructions involving the re-distribution of finances and goods and therefore they are highly political. Changes in eligibility criteria decide on the rise and fall of services, treatments and professions. On the other hand, eligibility procedures themselves are subject to availability of services and views of professionals, parents and advocates. Lobbying by such interest groups targets eligibility criteria or even diagnostic criteria to improve services for the persons they represent. Such groups can bring about changes in legislation and influence the way eligibility is established. For example, in Ireland, the influence of the Irish Society for Autism and successful litigation of parents of children with autism spectrum disorders led to the incorporation of this category into the 1998 Education Act and the establishment of a “Task Force on Autism” [20]. Other less fortunate groups may not benefit from such powerful representation.

### Means and ends of education

The means and ends of education have been discussed for generations by philosophers, policy makers and educators. Still today it remains a much debated topic in policy and practice. Compulsory education was extended to children with disabilities about forty years ago and since then, special educational services have been developed to ensure that all children benefit from education. But increasingly so, special education has been suspected of mainly fulfilling the selection function in education systems as postulated by disability advocates.

The United Nations Convention on the Rights of the Child requests and national legislations confirm that all children have a right to education. This right is explicitly extended to include children with disabilities as stated in many policy documents, most recently in the CRPD, Article 24. Within the broader framework of the general aims of education, the convention exemplifies the aims that are of special concern for children with disabilities (Article 24):

“(a) The full development of human potential and sense of dignity and self-worth, and the strengthening of respect for human rights, fundamental freedoms and human diversity;

(b) The development by persons with disabilities of their personality, talents and creativity, as well as their mental and physical abilities, to their fullest potential;

(c) Enabling persons with disabilities to participate effectively in a free society.”

Within the larger framework of a society and culture, education systems develop their own goals or student performance standards. Societies require the state to provide an education system and to implement processes that ensure these aims are met. Education systems are made up of policies, institutions and people operating at the national, intermediate and grass-roots level. All should contribute to establish and maintain processes that ensure that children with disabilities can make the best possible use of their right to education. As the Convention further demands, states should ensure that:

“(a) Persons with disabilities are not excluded from the general education system on the basis of disability, and that children with disabilities are not excluded from free and compulsory primary education, or from secondary education, on the basis of disability;

(b) Persons with disabilities can access an inclusive, quality and free primary education and secondary education on an equal basis with others in the communities in which they live;

(c) Reasonable accommodation of the individual’s requirements is provided;

(d) Persons with disabilities receive the support required, within the general education system, to facilitate their effective education;

(e) Effective individualized support measures are provided in environments that maximize academic and social development, consistent with the goal of full inclusion.”

National legislations are slowly aligning themselves to meet these aims by adopting bio-psycho-social frameworks of disability and developing new tools for assessment, changing educational planning and intervention, reforming teacher training and installing accountability systems to monitor progress. It is within this complex context that eligibility procedures are designed and implemented. According to the CRPD, access to inclusive education is to be granted without the requirement to meet any eligibility criteria. But if for some children special measures are necessary to ensure access or facilitate participation and if providing reasonable accommodation or individualized support measures requires additional resources, education systems will need to establish eligibility and define thresholds.

### Eligibility and the principles of the CRDP

An eligibility procedure compatible with the principles of the CRPD needs to address two issues separately: (1) establishing who falls within the definition of person with “disabilities” and (2) establishing this “individual’s requirements” such as for accommodation, support, or environments that maximize academic and social support and that are consistent with the goal of full inclusion. In addition, such an eligibility procedure needs to be based on a conceptual continuum from identification to intervention [21].

The CRPD defines persons with disabilities as follows: “Persons with disabilities include those who have long-term physical, mental, intellectual or sensory impairments which in interaction with various barriers may hinder their full and effective participation in society on an equal basis with others” (Article 1). The ICF is seen as the framework and classification adequate to represent the information required to use this definition [22,23]. It is better suited to describe the situation of children with disabilities than traditional, categorical approaches which are still used in many education systems to identify children with disabilities and for eligibility purposes (for an overview of definitions see OECD 2005, 28ff.) [24].

The ICF, like its predecessor, the ICIDH, has been suggested as a policy framework and therefore as a candidate for eligibility for its multidimensional functional properties and its sensitivity to environmental factors [25-30]. The ICF is able to represent multidimensional information and therefore holds the promise of a non-reductionist evaluation relevant for all types of services [10]. By focusing on functioning, information relevant to describe impairment and information relevant to describe participation in education can be bridged and considered in the same framework. Participation has been identified as the most important overall outcome for children and their families and its measurement should be further developed [31]. In addition, it is important to acknowledge that information not directly associated with an impairment or a syndrome may be most relevant to successful service provision in education [32]. Information used for eligibility purposes should therefore not only focus on functional characteristics that arise from a health problem.

To understand an “individual’s requirements” in a given educational setting, the situation of a child with disabilities needs to be linked to the participation prerequisites of education as a specific environment, a life area and an intervention setting. Diverse requirements such as adaptation, special assistance, additional services or attitudinal changes in teachers have been summarised under the term “need”. Since the 1990s, the term “special educational need” (SEN) has replaced the notions that children with disabilities require an altogether different education from children without disabilities. With the Salamanca Statement and Framework for Action in Special Needs Education the term “special need” has become internationally accepted referring not only to children with disabilities but also to gifted children and “children from linguistic, ethnic or cultural minorities and children from other disadvantaged or marginalized areas or groups” (UNESCO 1994, 6) [33]. While acknowledging that such special needs should be met in inclusive settings, the term fails to meaningfully distinguish between needs that arise due to participation restrictions that are known to be directly associated with impairment, participation restrictions associated with other child- or family-related factors, and participation restrictions due to specific barriers created by the school environment. “Special needs” are the result of the interaction of a child with a specific educational environment. Such environments are made up of behavioural and curricular expectations that do not necessarily respond to the needs of students. Problems in schools may also be created by schools and therefore eligibility should be linked to the context of teaching. The “response to intervention” approach seeks to account for this and some argue that an “assessment for intervention” is more adequate than an “assessment for determining labels” [34,35].

**Figure 1:**
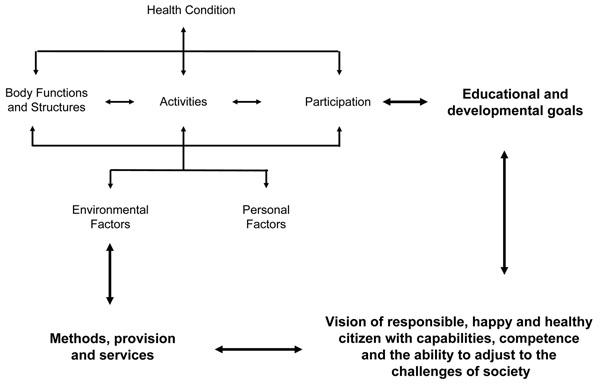
Expanded ICF model

Ultimately the policy context, financial resources and available services define which eligibility criteria are applied and how they are applied [36,9]. It is therefore unlikely that a standardised disability definition will lead to an equitable service provision independent of contextual influences as claimed by Tepper et al. [37]. An individual has to fulfil two conditions to gain eligibility: he or she has to be perceived as being “in need” of receiving the service or product in question and also “able to benefit” from it. It is not surprising therefore that policies for eligibility and service provision may vary dramatically [38]. Mehlman and Neuhauser suggest that “the best definition of disability may be the one that is the best predictor of something important” [39]. To predict the specific requirements of a child with disabilities in a given educational context, all relevant contextual variables should be considered. Different sources of information, such as the views of teachers and parents, need to be taken into account [40]. An important predictor of how much effort will be spent on the education of a child is the assumption of what that child may achieve: present participation is projected into the future and compared with the vision of capabilities, competences and abilities of a responsible, happy and healthy citizen. A definition of disability used for eligibility purposes in education systems should therefore take account of such views as they will influence efforts to help the child fulfil behavioural and curricular expectations. They will be reflected in the adaptations made to the environment and the time spent instructing the child. Therefore, the ICF model needs to be expanded to capture such envisaged aims (Figure 1).

## Results

### Defining and organising relevant information

When Switzerland was presented with the opportunity to change not only eligibility criteria, but also to develop a new procedure, all of the above-mentioned issues were taken into consideration. Formerly, eligibility was based on the premise that identified children were unable to participate in regular education. This implied referral to a special school or special setting. Eligibility was formally established based on the severity of impairment and explicitly excluded non health-related reasons such as social problems. In addition to transfer to a special school, eligibility could also provide access to additional interventions such as speech therapy or necessary services such as transport or housing. A list of birth defects and impairments was available, but no clear criteria were provided, resulting in a diverse practice across assessors, assessment centres and Cantons. There was wide dissatisfaction with this system as it encouraged perceptions of individuals’ differences rather than population diversity, focussed on impairments rather than functioning relevant for learning, and promoted segregation rather than inclusion. Whilst it is easy to acknowledge the need for such conceptual changes in principle, it is far more difficult to change the practices and attitudes of the individuals involved. The involvement of practitioners, disability advocates and parents was therefore given high priority. The conceptual and empirical work was monitored both by a group of expert and a group of stakeholders. Conferences and workshops were held at the different stages of the work process to ensure feedback from the widest possible audience in all regions of Switzerland.

In a first stage of the project, former eligibility procedures were disaggregated conceptually using the ICF-CY extended model as introduced in the last chapter. This work was carried out on the premise that disability is a multi-dimensional phenomenon involving not only the child but also its environment, and that establishing eligibility includes implicitly or explicitly postulating goals, interventions and needs. Disaggregation was done systematically using the following information components: (1) information on functioning, including body functions and activity/participation; (2) information on environmental factors, including both the professional and family environment; (3) information on diseases and other forms of categorical representations, using ICD-10 codes, former eligibility criteria and other problem descriptions; (4) recommended professional environments, including educational settings, specialised interventions; (5) recommended educational and developmental goals; and (6) estimation of requirements and needs.

These information sections were used to discuss conceptual issues and current practice. In addition, they were used as a framework for a data collection exercise involving 143 professionals responsible for eligibility in their respective services and Cantons. An electronic tool was developed in which participants entered relevant information of 400 children undergoing the traditional process of establishing eligibility. They were requested to reorganise information generated by them into the following dimensions: case history, family and school environment, functioning (list of items from body functions and activity/participation), diagnosis (ICD-10), assessment of individualised vs general educational goals, assessment of level and type of needs/requirements, recommendation of intervention or service provision. In addition, participants were also required to provide information on the assessment procedures they used and on the reasons for referral of the child.

A two-day training course provided an introduction to the conceptual framework and the more technical aspects of the electronic tool and set out the guidelines for mapping existing information into the new framework. Discussions were held on assessment strategies that practitioners had developed around the former eligibility criteria. There was a broad consensus that “disability” was not just an input variable, but may be at least partially the outcome of educational processes. The child’s response to interventions therefore should be considered when substantiating eligibility [35].

### Procedural Considerations

Based on these discussions, information components were classified into two parts, namely (a) information pertaining to functioning and disability as understood by the ICF-CY (basic assessment), and (b) information pertaining to requirements as conceptualised in the expanded ICF-CY model (assessment of requirements). The traditional approaches to eligibility tend to combine identification with treatment; the child is assigned to an intervention group. This leads to the dilemmas of difference described by Norwich [41] “The dilemmas of difference are found specifically in relation to core questions within education: i. whether to identify individual children as having SEN or a disability in the first place; ii. what children should learn – the curriculum; iii. where children should learn and with whom.” By detaching basic assessment information from considerations related to requirements and needs, the issues raised by Norwich can be addressed. These dilemmas can be resolved if they are tackled in individual cases, but not by prescribing fixed and pre-defined solutions for all. With the abandonment of such criteria, procedural considerations gain importance.

An important step in defining procedural guidelines was the agreement that not all information used to establish eligibility is of the same nature. There is a significant difference in documenting age, hearing loss and participation. Therefore different rules or standards should be applied for the generation of different types of data. The procedure contains information (Type 1) that is independent of contexts and can be generated by a specialist in a clinical setting, e.g. diagnosis of a disease and establishment of an impairment (ICD-10, body functions). This information is context-free, e.g. it is valid independent of the family situation or the current life situation of the child. Other information (Type 2) can hypothetically be generated by one person, but is dependent on temporal and spatial dimensions of specific life situations [31]. The ability to learn, for example, can only be observed in situations where learning occurs. Such context-specific information, e.g. on activities and participation or on environmental factors, can only be validly assessed if a variety of data from different occurrences or specific settings is compared and validated. While information types 1 and 2 can be objectively assessed (both used in the basic assessment module of the procedure), some information (Type 3) depends on contextual factors, such as the availability of resources, cultural values, perceived treatment priorities or prognosis on future functioning of the child, to name just a few.

Based on the above-mentioned typology of information, it was accepted that parents and teachers are not only important information sources, but that their views need to be reflected in the assessment of needs (Type 3). The Article 24 of the CRDP was brought to the attention of all stake-holders by pointing out that the eligibility procedure needs to address the right of the individual child to the best possible education and support. To address the dilemma of meeting universal human rights with limited resources, Bickenbach suggests that negotiation is inevitable: “One of the ironies of the theory and practice of human rights is that although, rhetorically, universal human rights are said to be ‘non-negotiable’, in fact negotiation is the primary political mode of the realisation of human rights” [22]. A transparent decision-making process that actively involves parents and children in determining goals, interventions and requirements is therefore more consistent with the CRPD than the application of pre-defined criteria.

Establishing eligibility by respecting the principles of the human-rights approach of the CRPD therefore requires special care in ensuring procedural justice. In education, there is never a single method, setting or goal that can be identified as superior over others; it is always about finding the best solution in a complex set of conditions and with limited resources available to do so. By making recommendations to individualise aims and means, the ideal of absolute equality and total inclusiveness is abandoned. It is also an acknowledgment that not enough was done for this child so far, that some things went wrong or expectations need to be adjusted. If eligibility is no longer simply reduced to describe a deficit of the child, all persons involved are exposed to uncertainty, potential incrimination and guilt. Where parents or teachers are seen as contributing factors to the participation difficulties of a child, this has to be made transparent, if such information is relevant for establishing eligibility. By designing a fair and transparent decision-making process that is documented adequately, consolidated recommendations can be put forth to an independent body. This central “clearinghouse” assumes the responsibility of a gate-keeper by comparing different cases and approving recommendations.

## Conclusions

This procedure was developed based on the premise that disability is a multidimensional phenomenon and the result of “the interaction between an individual and the individual’s personal and environmental factors” [42]. Functioning and disability are umbrella terms to encompass the multiple continua of functioning described from the perspective of the body, the individual and the society [1]. With the help of the ICF-CY model and classification, an inclusive and coherent framework to conceptualise low-incidence disabilities, high-incidence disabilities, learning or behavioural difficulties due to social disadvantages, and “normal” functioning has been developed. Children with disabilities are not a pre-defined special group with pre-defined special needs. Considering overall human functioning, there is more “sameness” of functioning with all children than “difference”. Education systems that respect the idea of a continuum of functioning should also offer a continuum of services based on the idea of personalising education [43]. All children have similar needs for relatedness, autonomy and competence [44] when engaging in learning. Participation in education therefore should be the first and most important concern when planning changes in educational settings and services. The items on functioning included in the procedure therefore do not try to fully describe all functional limitations; they were selected for their relevance in explaining differences in the capacity to participate in typical educational programmes and settings.

In addition, special or individualised goals can only be defined in the context of the general goals and aims of education as reflected in national curricula, student performance standards, or in the CRPD. The procedure takes this into account by requesting that a comparison be made between general goals (represented by the life domains of the ICF) and present functioning (represented by the list of activities and participation).

“Inclusiveness” is accepted as a principle of school organisation and curriculum, but it is insufficiently reflected in concepts of disability for the purpose of education. Often “exclusiveness” is more sought after by professionals and advocacy groups alike. Peters traces such conflicting language even in international policy documents related to “Inclusive Education” or “Education for All” [45] and it can be expected to note such discrepancies and conceptual contradictions also in national documents. Educational policies and practices focus on the interaction between individuals and society, vacillating between qualifying all children to their best abilities and preparing children for different social roles and positions. Not only in Scotland, as reported by Tisdall and Riddell [46], but also in many other policy contexts relevant for children with disabilities, different strategies and discourses are competing with each other. Politicians and policy makers increasingly express their commitment for inclusion and social justice, but considerable obstacles remain and for some groups. Exclusion rates for some groups have actually increased over the last few years [47]. Agendas on “raising standards” and a “commitment to choice, diversity and competition” are seen by many as inherently antithetical to inclusion [48]. The need to discuss and clarify different values, preferences and goals is taken into account by introducing the notion of a typology of information that acknowledges that means and ends of education are debatable and therefore need to be negotiated.

Using the ICF-CY model to identify and organise different components and types of information facilitates the comparability across disabilities and settings. This is an important prerequisite for a transparent and equitable distribution of resources. The procedure generates data that helps understand how thresholds are set and which differences in the individual or the school and family environments are associated with individualised goals or special interventions.

The implementation of the procedure will provide an opportunity to introduce a more meaningful and comparable instrument to represent information as well as a new approach to decision-making. In addition, the procedure as it exists now (definition and description of information components of the procedure, a handbook and an electronic tool) will be further developed in a coordinated process together with practitioners, policy makers and disability advocates. The data generated during the implementation phase will be used to further improve the list of selected ICF items. It is also planned to develop standards for mapping assessment procedures onto the ICF-CY. This standardisation process has to take account of current practices as well as general principles [49] to develop linking rules that are consistent and at the same time meaningful to practitioners. Data generated during the implementation phase will also be used to better understand gate-keeping mechanisms. These analyses will provide a unique opportunity to initiate an evidence-based debate on matters of inclusion, participation, service provision and the goals of education.

## Competing interests

The authors declare that they have no competing interests.
